# Relation between Exercise Performance and Blood Storage Condition and Storage Time in Autologous Blood Doping

**DOI:** 10.3390/biology10010014

**Published:** 2020-12-29

**Authors:** Benedikt Seeger, Marijke Grau

**Affiliations:** Molecular and Cellular Sports Medicine, German Sport University Cologne, 50677 Cologne, Germany; benediktseeger@web.de

**Keywords:** autologous blood doping, exercise performance, storage conditions, storage duration, red blood cells

## Abstract

**Simple Summary:**

Autologous blood doping (ABD) refers to sampling, storage, and re-infusion of one’s own blood to improve circulating red blood cell (RBC) mass and thus the oxygen transport and finally the performance capacity. This illegal technique employed by some athletes is still difficult to detect. Hence knowledge of the main effects of ABD is needed to develop valid detection methods. Performance enhancement related to ABD seems to be well documented in the literature, but applied study designs might affect the outcome that was analyzed herein. The majority of recent studies investigated the effect of cold blood storage at 4 °C, and only few studies focused on cryopreservation, although it might be suspected that cryopreservation is above all applied in sport. The storage duration—the time between blood sampling and re-infusion—varied in the reported literature. In most studies, storage duration might be too short to fully restore the RBC mass. It is thus concluded that most reported studies did not display common practice and that the reported performance outcome might be affected by these two variables. Thus, knowledge of the real effects of ABD, as applied in sport, on performance and associated parameters are needed to develop reliable detection techniques.

**Abstract:**

Professional athletes are expected to continuously improve their performance, and some might also use illegal methods—e.g., autologous blood doping (ABD)—to achieve improvements. This article applies a systematic literature review to investigate differences in the ABD methods and the related performance and blood parameters owing to different storage conditions—cryopreservation (CP) and cold storage (CS)—and different storage durations. The literature research resulted in 34 original articles. The majority of currently published studies employed CS during ABD. This contrasts to the applied storage technique in professional sports, which was mainly reported to be CP. The second outcome of the literature research revealed large differences in the storage durations applied, which were in the range of one day to 17 weeks between blood sampling and re-infusion, which might affect recovery of the red blood cell mass and thus performance outcome related to ABD. Data revealed that performance parameters were positively affected by ABD when a minimal storage duration of four weeks was adhered. This article identified a need for further research that reflect common ABD practice and its real effects on performance parameters, but also on related blood parameters in order to develop valid and reliable ABD detection methods.

## 1. Introduction

International world class athletes are expected to continuously improve their performances [1,2] and some athletes not only use legal but also illegal methods to achieve this improvement [2,3]. Autologous blood doping (ABD) appears to be a widely applied illegal method to enhance an athlete’s performance [4,5]. The reason why ABD represents a preferred method might be due to the finding that the muscular oxygen (O_2_) supplying system is a major limiting factor in endurance sports [6] and ABD is able to increase this system without being detectable so far [7,8]. This assumption is further supported by the recent disclosures and the ongoing doping investigation and trial in Germany referred to as “Operation Aderlass” [9,10]. However, since doping does not only harm the sport’s reputation [11], but also possibly the athlete’s health [12,13], it is important to develop a reliable and valid ABD detection method. This systematic review aims to analyze published protocols of applied ABD techniques and the related effects on tested performance parameters in order to evaluate the effectiveness of the methods applied, especially related to changes of the in vivo red blood cell (RBC) system, and to compare the applied methods with actual doping practice. This knowledge will be needed to assess the real effects of published ABD-related protocols in order to understand the related changes in the RBC system necessary for the development of ABD detection methods.

The ABD procedure consists of three major steps: (1) the blood sampling from a subject, (2) the processing and storage of the blood or blood components, and (3) the re-transfusion of whole blood or a red blood cell (RBC) concentrate to the initial donor [4]. Each of these steps can be implemented with some variations, which in turn might affect the effect size of ABD [4]. First, the donated blood volume (BV) can vary [12]. Second, two different storage techniques, cryopreservation (CP) or cold storage (CS), are applied [14]. Thereby, CP refers to blood storage between −65–−140 °C, while CS refers to blood storage at 4 °C, which is equivalent to refrigerator level [14,15]. The storage techniques will be explained in more detail in Section 3.1, when they are discussed. The storage duration might differ between the storage techniques since CS stored blood is durable for 35–42 days, while CP allows a blood storage for up to ten years [12,16].

The blood preparation and storage methods of the blood have not been compared so far. This appears to be a research gap, because the restoration of RBC mass after sampling is an important factor for the effect size of ABD. In addition to that, the storage technique might influence the quality of the re-infused RBC. It is widely agreed that an increase in RBC and a corresponding increase of the hemoglobin mass (Hb) is the major determinant of ABD-related performance enhancements [3,12]. ABD might affect RBC structure and/or function, which might affect exercise performance [17,18], but the precise mechanisms related to the different ABD methods remain to be investigated Third, according to current literature, the amount of re-infused blood after storage varies between 135 and >900 mL, but a possible dose response relation of re-transfused blood and performance parameters was not described so far [19,20].

The aim of this review was to analyze and compare the current literature regarding reported differences in the storage conditions and the storage duration applied during ABD and to assess the provided changes in exercise performance in relation to the study design. This review also aimed to direct the focus on RBC changes caused by the ABD techniques. This information may provide new starting points for anti-doping advocates in the development of ABD detection techniques.

## 2. Method

**Figure 1:**
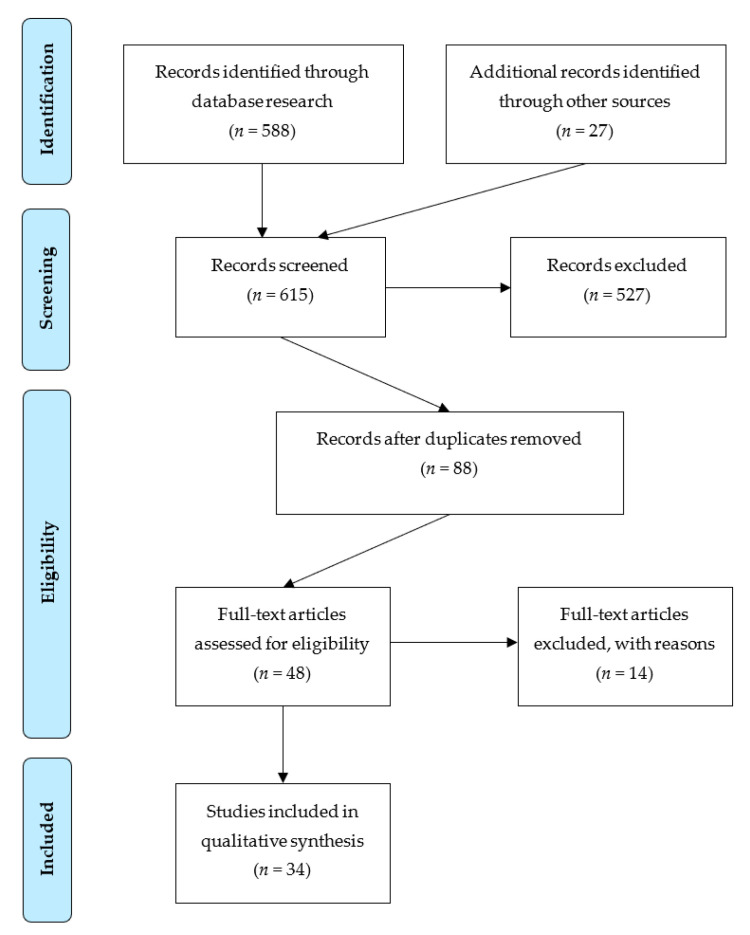
Research process. Flow diagram inspired by PRISMA 2009 Flow Diagram [21].

A systematic search protocol was constructed that followed the PRISMA guidelines for systematic reviews to ensure the reproducibility of this systematic literature search [21]. This procedure is also recommended by the MDPI [22]. The research process is presented in a flow diagram (Figure 1) inspired by the PRISMA 2009 Flow-Diagram [21]. However, since no meta-analysis was conducted, the final step of the PRISMA 2009 Flow Diagram “Studies included in quantitative synthesis” was not carried out.

The primary aim, and thus end point, of this review was to evaluate described effects of blood re-transfusion on exercise performance with a special focus on the described storage technique and storage duration and to relate these changes to possible changes in the red blood cell system.

To select articles, that are eligible for the reviewing process, a full text search was conducted. Applied keywords included “autologous blood doping” and “autologous blood transfusion” combined with either “sport”, “exercise”, or “performance”—using the fixed Boolean operators of each database. The used databases included PubMed, BioMedsearch, Spolit, Web of Science, EBSCOhost, and ProQuest. The inclusion criteria for articles of these databases were: (1) articles in peer reviewed journals, (2) the reference to a sport context, (3) articles investigating blood parameters and/or exercise performance, and (4) the accessibility of the full text. No restrictions concerning the publication dates were made. Articles that fulfilled the inclusion criteria in title and abstract were taken into the record. The first step in the research process led to *n* = 588 articles. Additionally, a forward and a backward search was conducted for the identified articles. The search engine Google Scholar (scholar.google.com) was used for these searches. The forward backward search resulted in additional *n* = 27 articles, thus a total amount of *n* = 615 articles was identified for the screening process. During the screening process, *n* = 527 articles were removed as they did not meet the review’s criteria. The screening resulted in *n* = 88 articles for further research. After removing duplicates, a full text examination of the remaining *n* = 48 articles was conducted to identify the articles qualified for the qualitative analysis. During this step, review articles were removed too, since they do not present primary data (they were included in the search as they are part of the literature and are eligible sources to identify applicable articles). The final search process resulted in *n* = 34 articles.

The analysis of the selected articles focused on two major aspects. First, the blood storage technique applied (CS or CP) were described and compared regarding possible differing effects of CS and CP on changes of performance and related blood parameters. Second, the time interval between blood donation and re-infusion was analyzed. The effect of improved performance after re-transfusion of stored blood relates to the restoration of initial RBC mass and the additional benefit of extra blood volume after the re-transfusion. The articles were analyzed whether or not a full recovery of RBCs might be reasonable, the minimal period between donation and re-infusion to gain an effect on exercise performance and if the length of the period affects the ABD outcome.

## 3. Results and Discussion

ABD is applied in order to increase RBC mass and thus oxygen transport capacity of the blood and to improve exercise performance [23]. ABD not only increases oxygen delivery to the working muscle but also increases the heat tolerance of the athletes [24]. The International Olympic Committee (IOC) banned blood boosting after the 1984 Olympics [25]. Flow cytometry methods for membrane surface double population of antigens may reveal the homologous blood transfusion approach [26,27,28] but autologous blood transfusion is not detected by this method. Thus, new approaches are designed as indirect markers for blood doping, including total Hb mass measurements, or to test for the excretion of metabolites of bag plasticizers in the urine. Another attempt to detect autologous transfusions is the Athlete Biological Passport (ABP) [29,30] which contains the longitudinal monitoring of biologic measures to identify patterns that might be related to ABD and the evaluation of such abnormal patterns by a panel of experts. These parameters might be affected by the storage duration and storage technique via an effect on RBC. Parameters include RBC count, Hb concentration, hematocrit, mean cellular Hb concentration, mean cellular volume, and the reticulocyte percentage [31,32]. Further, OFF-Hr score ([Hb]—60√ Ret%, normal range: 85–95 [7]) and abnormal blood profile score (ABPS)—which are calculated parameters and are also mainly dependent on RBC related parameters—are also monitored in the ABP [33,34]. This strategy might involve several drawbacks, including differences in threshold values between the different sports associations and the difficult interpretation of hematological parameters because of wide inter-individual differences [35]. Recent data suggest that the RBC function itself might be affected by the ABD process and that the measurements of certain RBC parameters, for example RBC deformability, might in the future provide a promising attempt to detect ABD [17,36]. Still, valid detection methods are unavailable at present, but might benefit from the knowledge on the real effects of performance improvements after ABD and the relation to changes in RBC or Hb concentration.

As mentioned earlier, ABD is capable of improving the muscular O_2_ supply system and thus endurance exercise performance. Several studies analyzed within this review reported either improvements in the VO_2max_ or VO_2peak_, or augmentation of the time to exhaustion test (TTE) or the time trial test (TT). VO_2max_ refers to the maximum possible O_2_ availability (VO_2_) for the working muscles. VO_2_ peak is provided if the monitored oxygen uptake values do not flatten during the exercise test but still increase at time of termination of the exercise test [37,38]. Thus, both tests are considered to be the most adequate to examine a subject’s endurance capacity [37,39]. Because VO_2max_ and VO_2peak_ are often used incorrectly as synonyms [40], this review will not differentiate between theses parameters. The TTE measures the time a subject is able to perform a standardized exercise protocol [41]. The TT quantifies the time a subject needs to finish a standardized task, or the standardized work a subject is able to complete within a fixed time frame [42]. The findings of improvements in endurance exercise due to ABD were in line with the current literature [3,12,19].

The wide range of exercise improvements is probably related to differences in the implied ABD method. Different storage technique and varying storage durations appear to be a moderator to the ABD outcome. Therefore, the following section aims to outline differences in the applied ABD—concerning storage duration and storage technique—and how these affect performance and performance related blood parameters. Therefore, Section 3.1 and Section 3.2 each discuss a part of the ABD method. To gain an optimal understanding, Table 1 serves as an overview of the applied methods and important parameters of the selected articles.

### 3.1. Blood Storage Techniques

The performed literature analysis suggested two different storage techniques applied during ABD: CP and CS.

ABD involves sampling of 1–4 units of blood (1 unit corresponds to 450 mL blood) 8–12 weeks before competition to allow recovery of the RBC mass back to pre-donation level. The blood is centrifuged, the plasma is usually transferred back to the donor or discarded, and the RBC are stored and re-infused into the donor/athlete 1–7 days before a competition [12]. During CS, RBC were stored in the storage solution SAGM (saline, adenine, glucose, mannitol) at 4 °C, but several published articles suggest that the quality of RBC decreases during storage at 4 °C and that re-infusion of long term stored RBC might be deleterious [75,76]. These deleterious effects are reduced when RBC are cryopreserved [36]. During CP, blood is sampled in common blood bags and anticoagulated using CPDA-1 solution (citrate, phosphate, dextrose, and adenine). The blood bags are automatically processed to separate the RBC. Glycerol is added as cryoprotectant [15,77] and the RBC are either fast frozen (>100 °C/min) and stored below −140 °C or slowly frozen (~1–3 °C/min) and stored between −65 and −80 °C [15,77]. The hypothermia of the CP stops the aging of RBC and thereby prevents the maturity of the cells [36], while the 4 °C of CS only slows the aging but does not prevent the cells death [78,79]. Cryopreserved RBC are thawed when needed by an automated cell processor device [12]. Differences between CS and CP include the allowed storage time and the occurrence of the so-called storage lesions [12]. Maximum storage time of CS ranges between 35 and 42 days, while cryopreserved RBC are allowed to be stored up to 10 years [12,14]. Further aspects of the storage durations will be addressed in Section 3.2. Blood storage is associated with the loss of RBC. According to recent literature, the RBC loss in CS is about 6% per week, while the loss of RBC during CP is stated to be about 15% in total due to the processing for the CP storage [14].

From the 34 selected articles, 11 studies used CS while 15 articles used CP, another three articles used both techniques and five articles did not mention their storage technique. Improved performance parameters and/or increased associated blood parameters were presented by nine out of 11 (9/11) CS studies, 14/15 CP studies, and 2/3 CS+CP studies. Thus, it seems plausible that improvements of the performance might be achieved by both storage techniques [20,48,53]. It was not possible to argue whether there is a clear detectable relation of the performance increases and a specific storage technique because the selected studies showed high variations in their study designs. Only Celsing and co-workers [49], Lamberti and colleagues [57], and Mørkeberg and associates [16] applied both storage techniques at the same time. But the study by Celsing et al. [49] mixed both storage techniques within one experiment and thus a comparison between the techniques was not possible. Furthermore, Lamberti and co-workers did not differentiate between CP and CS in their results and did not present any data to compare the storage techniques. In contrast, Mørkeberg and colleagues differentiated between Hb level changes in CP and CS. The authors detected a 3.6% increase in Hb content after re-infusion of blood stored under CS conditions and an increase in Hb level of 6.5% after the re-infusion of blood stored under CP conditions (both groups were re-infused with “all three bags” [16] (p. 287). This might suggest a higher increase in Hb concentration by CP. However, the study design implemented a time frame of 4 weeks between blood sampling and re-infusion for the CS group and a period of 10 weeks for the CP group. Because this difference might affect the recovery of RBC amount and Hb mass in vivo, the results were not suitable to compare both storage techniques.

The data suggested that possible differences between CS and CP in performance and related blood parameters appeared to be less understood but relevant because the storage condition and the known quality changes potentially affected the results described. Further, the quality of the stored RBC was affected by so called storage lesion, which differs between CS and CP. The precise findings of storage lesions will not be addressed in this review and were described in detail elsewhere [80,81]. However, some aspects will be described herein. RBC lose their deformability, an important parameter to ensure nutrient and oxygen supply in the microcirculation, during CS [82], while RBC deformability of cryopreserved RBC appears not to be negatively affected [36]. The decrease in RBC deformability in CS might be associated to RBC senescence, which includes not only a reduction in cell volume, externalization of phosphatidylserine, and reduction in CD47 [83], but also a loss in 2,-3-DPG and ATP and reduction in nitric oxide bioavailability. During senescence in CS, cell volume loss stiffens the RBC and leads to reduction in RBC deformability [84]. Thus, old RBC are prone to hemolysis or phagocytosis during the circulation and might reduce the outcome during re-infusion [75]. Stopped or highly reduced RBC ageing during CP storage is thus of advantage to preserve the quality of the cells. Further, the deglycerolization procedure applied during the thawing of CP cells and mainly responsible for the RBC loss during CP is suggested to destroy mainly old RBC [85]. Hence, the average RBC population age is reduced in CP in comparison to CS. This further positively affects the RBC deformability, density, size, and O_2_ affinity [18,84], which leads to an increased diffusion capacity and an improved muscular O_2_ supply [18,86]. Since there appear to be several differences in the RBC count, Hb content concerning the different storage techniques, it appears to be relevant to investigate these differences directly.

Furthermore, it is noteworthy that the majority of the articles published in the last 20 years either implied CS [19,20,43,60,61,64] or do not comment on the storage technique [87]. This appears to be contradictory to the actual applied ABD techniques in professional sports [11,88]. Because the different storage techniques in ABD probably lead to different blood adaptations and possibly different enhancements of the endurance performance, there is a need for future studies. These should focus on the effects of CP on functional blood parameters in relation to endurance capacity. This knowledge will allow to identify parameters affected by this procedure in order to develop new detection methods.

### 3.2. Blood Storage Duration

The second variable affecting the outcome of the ABD method is the storage duration. This refers to the time period between the blood donation from a subject and the blood re-infusion back to the initial donor. The importance of this phase during ABD is due to the fact that the increase in RBC mass after re-infusion evolves not from the re-infusion itself, but from the interplay between the recovery of RBC mass in vivo after sampling [89,90]—caused by an artificially created momentary anemia [91,92,93]—and the following re-infusion of additional RBC [3,12]. A decrease in circulating RBC after blood sampling increases erythropoietin (EPO) expression in the kidney and release, which stimulates erythropoiesis in the bone marrow [91]. The production of RBC starts with a pluripotent hematopoietic stem cell [89,90]. Among others, EPO is the key driver for of the differentiation of the progenitor cells into colony forming units-erythroid, proerythroblasts, and erythroblasts [94,95]. Erythropoiesis lasts approximately five to nine days. Reticulocytes are then released into the circulation to mature into RBC [90].

The RBC restoration of the RBC mass to pre-donation level [96] is essential for the performance enhancement caused by ABD. An additional aspect of the increase in erythropoiesis rate after blood sampling is a probable change in the RBC quality. The neoformation of young RBC reduces the average age of the circulating RBC pool [17]. This reduction in RBC age results from the interplay of an increased RBC production accompanied by a normal RBC mortality rate [17,97]. Young RBC are more deformable compared to other RBC and are suggested to show increased oxygen transport capacity [84,86]. Thus, rejuvenation of circulating RBC might improve ABD effect size [18]. However, further studies are needed to support this relation.

Although the restoration period appears to be a central element in ABD, only two articles tested the effect of different storage durations on exercise performance [51,58]. However, one of the two studies [51] tested differences in sampled blood volume in parallel and thus, the results of this article were less suitable for a comparison of storage durations. The second study [58] tested the exercise performance of the candidates after re-transfusion of the blood after a storage duration of 15 weeks and 2 weeks, respectively. After a storage duration of 15 weeks, TTE increased by 15%, VO_2max_ by 17%, and also RBC count and Hb were significantly increased. In contrast, after 2 weeks of storage, Hb level and RBC count were not significantly altered. A performance test was not carried out [58]. These data indicate that the storage duration should be long enough to allow the blood parameters to reach the initial level prior to re-transfusion of the stored blood in order to significantly affect exercise performance [58].

According to current literature, recovery of the RBC content after donation of 500 mL of whole blood is estimated to last 59 days [60,74,98]. Referring to the selected articles in this review, only Robertson and co-workers [62] respect this recovery period. However, studies reporting a shorter period between blood donation and re-infusion present improvements in endurance performance and related blood parameters [20,51,74]. In general, the selected articles present a wide range of time frames between blood donation and re-infusion of 1 day up to 17 weeks [61,62]. The analysis of these articles revealed that the minimal recovery period needed to establish significant improvements in the endurance performance or relevant blood parameters appeared to be 4 weeks. Nine out of thirty-four articles described a storage time of less than 4 weeks (1 day to 3 weeks). However, only four of these articles detected significant improvements in TTE of up to 40% [43,56,63], physical working capacity (PWC) of 3.9% [54], Hb level of 14.3%, and RBC of 14.8% [56]. These findings suggest that this rather short recovery period might be sufficient for the tested subjects to at least recover a part of their RBC mass after sampling and that this might be sufficient to improve the performance. However, the recovery process of RBC is rather complex and mainly depends on an increased erythropoiesis, which might take one week to produce additional RBC [99]. Therefore, full recovery of the whole RBC mass during a recovery time of three weeks seems unlikely. Further, three of the four mentioned articles [43,54,63] sampled a blood volume >500 mL, which might need even more time to recover than the reported 59 days. The three articles that reported improvements in exercise performance after a recovery time of less than three weeks showed some limitation in the study design. The authors scheduled the baseline test after the blood sampling, shortly before the re-infusion [43,56,63]. Thus, baseline values of the performance parameters, but also RBC count and level of Hb, might be artificially reduced because of the blood loss. The increase in performance parameters likely express the effect of a restoration rather than an “on top” effect that is intended. Thus, a central element of the ABD mechanism—the need of RBC mass recovery—was disregarded. In contrast to that, Gullbring and co-workers [54]—the fourth article detecting an increase in endurance performance with storage durations less than four weeks—performed the baseline measurements before the blood sampling. PWC performance significantly increased by 3.9%. in the post re-infusion test. However, it cannot be ruled out that this result might also be related to an adaptation to the test protocol because the authors scheduled several tests between sampling and re-transfusion as well.

Furthermore, the test performed one day pre re-infusion presents a similar PWC performance compared to the test 1h post re-infusion. In addition, no significant increase in the Hb level was detected by the authors. This missing Hb increase presents a further indicator that the performance increase might be rather related to muscular adaptation than to changes in the subjects’ blood parameters. In contrast to the aforementioned articles, five of the articles analyzed herein with a storage duration of less than 3 weeks did not report any significant improvements in the tested performance—or relevant blood parameters. Furthermore, all articles with a storage duration of 4 weeks or more present significant changes in either the endurance performance, relevant blood parameters, or in both.

In summary, articles that described improvements in performance parameters after blood re-infusion but had storage durations less than four weeks also presented methodical weaknesses. Articles with storage durations less than four weeks and a reasonable study design regarding the exercise tests presented no significant improvements, but all articles with a storage time of 4 weeks or more, which might be needed to fully recover RBC mass, report improvements in performance and/or in related blood parameters including RBC or Hb. Thus, it was concluded that a minimum of four weeks is needed as RBC restoration time for any ABD effect on performance parameters. Future anti-blood doping studies should consider this information during the development of detection methods.

## 4. Limitations

The present article provides a better understanding of two key factors affecting the exercise performance related to ABD. Nevertheless, the small number of key words comprised in the applied search string might represent a limitation of the present review. For instance, in some articles the re-transfusion of RBC is also referred to as induced erythrocythemia. However, this term was not included in the conducted search, because it would also lead to articles not associated to autologous blood doping. Also, the literature search of this review only focused on peer reviewed original articles and thus excludes alternative sources such as proceedings, conference papers, and books [100].

## 5. Conclusions

This review revealed that the storage technique applied, and the storage duration adhered are two major factors that affect the endurance performance capacity in the context of autologous blood doping (ABD). The results of the literature review indicated that cold storage (CS) shows disadvantages compared to cryopreservation (CP) with regard to possible storage time. Also, the quality of RBC highly reduced during CS, which might lead to adverse effects for the exercise capacity. The review indicated that CP might be favored in the ABD procedure. The literature analyzed herein further revealed that a large proportion of published articles reported storage durations that might be insufficient to allow the restoration of initial RBC mass. This might in part explain the absence of positive effects of re-infused RBC on exercise parameters, but might also lead to the assumption that the reported positive effects of ABD on exercise performance might at least in part result from other influencing variables.

In order to understand the physiological changes in the RBC system during ABD, which is indispensable to develop ABD detection techniques, it might be of relevance to focus on three topics in future research: (1) investigation of the influence of CP on the ABD outcome because this might be the most used in current doping cases, (2) investigation of the influence of a proper restoration time on ABD outcome, (3) investigation of the changes in functional and structural RBC parameters during ABD.

## Figures and Tables

**Table 1:** Overview of analyzed original articles related to effects of blood storage techniques and storage duration on performance outcome in autologous blood doping (ABD).

Article	*n*	Fitness	Donated	Stored	Infused	TTE/TT	VO_2m/p_	RBC	Hb
Bejder et al. [20]	9M	ht	450 mL	CS 4 wk	136 mL	TT + 4.4% *	p/	+ 3.3% *	+ 2.9% *
				CS 4 wk	369 mL	TT + 5.1% *	p/	+ 9.8% *	+ 8.9% *
Bennett-Guerrero et al. [43]	4M	t	900 mL	CS 1 wk	900 mL	TTE + 8.4% *	m + 8.7% *		
	4M	t	900 mL	CS 1 wk	900 mL	TTE − 2.6% *	m + 1.9% *		
Berglund et al. [44]	8M+4F	t	3 × 450 mL	CP ~14 wk	n/a				F + 18% * M + 12% *
Berglund & Hemmingson [45]	6M/F	ht	1 × 900 mL + 1 × 450 mL	CS 4 wk	1350 mL	TT + 5.3% *			+ *
Berglund et al. [46]	6M/F	ht	1 × 900 mL + 1 × 450 mL	CS 4 wk	1350 mL	TT n/a			+ 7.9% *
Brien et al. [47]	6M	t	2 × 450 mL	CP	400 mL	TT + *			
Buick et al. [48]	11M	ht	1000 mL	CP	900 mL	TTE + 33% *	m + 5% *		+ 8% *
Celsing et al. [49]	9M	t	5 × 450 mL	CS + CP 1–9 wk	1800 mL		m/		
Celsing et al. [50]	8M	t	8 × 450 mL	5–7 wk	n/a		m/		
Ekblom et al. [51]	3M	ut	3 × 400 mL	6 wk	n/a	TTE + 23% *	m + 9% *	+ 18% *	+ 13% *
	4M	ut	800 mL	4 wk	n/a	TTE + *	m + *		+ 4.9% *
Ekblom et al. [52]	5M	t	800 mL	CS	360 mL		m + 8% *		+ 4.9% *
Goforth et al. [53]	6M	ht	2 × 450 mL	CP	330 mL	TT + 2% *	m + 11.9% *		+ 10% *
Gullbring et al. [54]	6M	ut	530–689 mL	CS 1 wk	n/a	PWC + 3.9% *			/
Kanstrup & Ekblom [55]	M	t	900 mL + 500/750 mL	CS	500/750 mL	TTE + 24% *	m + 2–11% *		+ 4% *
Kots et al. [56]	10M	ht/ut	500 mL	3 wk	500 mL	TTE + 40% *	m/	+ 14.8% *	+ 14.3% *
Lamberti et al. [57]	24M	t	450 mL	CS/CP 5 wk	n/a			+ *	+ *
Malm et al. [58]	10M	t	2 × 450 mL	CP 15 wk	n/a	TTE + 15% *	m + 17% *	+ *	+ *
	30M/F	t	1 or 2 × 450 mL	CP 2 wk	n/a			/	/
Mørkeberg et al. [16]	23M	t	3 × 450 mL	CS 4 wk	n/a				+ 3.6%*
				CP 10 wk					+ 6.5% *
Muza et al. [59].	12M	SF	2 × 450 mL	CP ~12 wk	600 mL		m + 11% *	+ 11% *	+ 10% *
Pottgiesser et al. [60]	10M	n/a	550 mL	CS 7 wk	280–350 mL			+ *	+ 5–8% *
Pottgiesser et al. [61]	11M	n/a	550 mL/1000 mL	CS 1 d	330–550 mL				− *
Robertson et al. [62]	9F	n/a	2 × 450 mL	CP ~9–17 wk	475 mL		m + *	+ *	+ *
Robinson et al. [63]	6M	n/a	1000–1200 mL	~2 wk	1000–1200 mL	TTE + *	m/		
Sallet et al. [64]	7M	ht	450 mL	CS ~3 wk	450 mL			+	/
Sawka et al. [65]	30M	n/a	450 mL	CP	600 mL		m + *		+ 10% *
Sawka et al. [66]	9M	t	2 × 450 mL	CP 6 wk	600 mL		m + 11% *	+11% *	+ 10% *
Spriet et al. [67]	4M	ht	1 × 900 mL + 1 × 450 mL	CP ~10 wk	450 mL		m/		+ *
	4M	ht			900 mL		m + 7.5–10.7% *		+ *
	4M	ht			1350 mL		m + 10–13.3% *		+ *
Thomson et al. [68]	4M	t	2 × 500 mL	CP ~12 wk	n/a	TT + 10% *	m + *		+ *
Thomson et al. [69]	4M	ut	2 × 500 mL	CP	n/a	TT + *	m + *		+ *
Turner et al. [70]	7M	n/a	2 × 450 mL	CP	n/a	TT + 5.3% *	m + *		+ 3.9% *
Williams et al. [71]	5M	ht	500 mL	CS 3 wk	500 mL	TTE/			
	5M	ht			275 mL	TTE/			
Williams et al. [72]	16M	ht	460 mL	CP 3 wk	460 mL	TTE/		/	/
Williams et al. [73]	12M	ht	2 × 460 mL	CP ~9 wk	920 mL	TT + *		+ *	+ *
Ziegler et al. [74]	8M	n/a	450 mL	~4 wk	245 mL	TT + 4.6% *	p + 4.8% *	+ *	+ *

Legend: VO_2m/p_ = VO_2max_/VO_2peak_; m = max; p = peak; RBC = red blood cell; Hb = hemoglobin; CS = cold storage at (4 °C); CP = cryopreserved; wk = week(s); d = day(s); M = male; F = female; ht = highly trained; t = active/moderately trained; ut = untrained; SF = special forces; TT = time trial test; TTE = time to exhaustion test; PWC = physical working capacity test; + = increase; / = no change; − = decrease; * = significant; X.X% = change in %; n/a = not available.

## Data Availability

Not Applicable.
